# Acute adrenal failure (AAF) as the presenting symptom of primary antiphospholipid syndrome (APS)

**DOI:** 10.1186/1546-0096-9-S1-P252

**Published:** 2011-09-14

**Authors:** G Russo, R Carlomagno, C Forni, L De Martino, D Capalbo, M Alessio

**Affiliations:** 1 Department of Pediatrics, Federico II University, Naples, Italy

## Introduction

AAF is an uncommon manifestation of systemic lupus erythematosus and a rare complication of APS. We report a pediatric patient presenting initially with AAF which preceded any other manifestation of the APS.

## Case report

FV previously healthy 11-year-old boy, developed abdominal pain and fever. An abdominal computed tomography scan showed nodular lesions in the adrenal glands. He was referred to our Department, where the diagnosis of APS and adrenal failure were considered on the basis of the following laboratory findings: positive antiphospholipid antibodies (IgG 20,9 IgM 27,3 n.v. <10), high plasma ACTH level (961 pg/ml n.v. 10-130), low plasma cortisol levels (31,5 ng/ml n.v. 50-200). Other data included anemia, thrombocytopenia, elevated inflammatory parameters, hypergammaglobulinemia, prolonged partial thromboplastin time, positive antinuclear antibodies, negative double-stranded DNA, anticardiolipin antibodies 56,6 U/ml (nv 0-20), positive lupus anticoagulant test and Coomb’s test, elevated renine activity 36,40 pg/ml (n.v. 1,3-16). MRI confirmed bilateral adrenal hemorrhage. A treatment with intravenous metylprednisolone, followed by oral prednisone and anticoagulant, was started promptly, resulting in progressive improvement. After 2 months he presented hyponatremia and elevated renine activity (>300 pg/ml), treated with mineralcorticoid replacement. After 1 year, because of thrombocytopenia, positive lupus anticoagulant test, antiphospholipid antibodies and Coomb’s test, cyclosporine was associated, with improvement of clinical conditions.

## Conclusion

The development of AAF has been rarely reported in APS due to adrenal hemorrhage following vascular occlusion of adrenal vessels. This case emphasizes the importance in the assessment of antiphospholipid antibodies in all patients with rapidly progressive AAF and concurrent abdominal pain.

